# Preparing, conducting, and analyzing Delphi surveys: Cross-disciplinary practices, new directions, and advancements

**DOI:** 10.1016/j.mex.2021.101401

**Published:** 2021-05-28

**Authors:** Daniel Beiderbeck, Nicolas Frevel, Heiko A. von der Gracht, Sascha L. Schmidt, Vera M. Schweitzer

**Affiliations:** a Center for Sports and Management, WHU, Otto Beisheim School of Management, Erkrather Str. 224a, 40233 Düsseldorf, Germany; b School of International Business and Entrepreneurship, Steinbeis University, Kalkofenstr. 53, 71083 Herrenberg, Germany; c Chair of Leadership, WHU, Otto Beisheim School of Management, Erkrather Str. 224a, 40233 Düsseldorf, Germany

**Keywords:** Clinical trials, Consensus method, Cross-impact analysis, Decision-making, Delphi method, Expert opinion, Foresight, Judgmental forecasting, Sentiment analysis, Scenario analysis

## Abstract

Delphi is a scientific method to organize and structure an expert discussion aiming to generate insights on controversial topics with limited information. The technique has seen a rise in publication frequency in various disciplines, especially over the past decades. In April 2021, the term *Delphi method* yielded 28,200 search hits in Google Scholar for the past five years alone. Given the increasing level of uncertainty caused by rapid technological and social change around the globe, collective expert opinions and assessments are likely to gain even more importance. Therefore, the paper at hand presents technical recommendations derived from a Delphi study that was conducted amid the outbreak of the COVID-19 pandemic in 2020.

• The paper comprehensively demonstrates how to prepare, conduct, and analyze a Delphi study. In this regard, it combines several methodological advancements of the recent past (e.g., dissent analyses, scenario analyses) with state-of-the-art impulses from other disciplines like strategic management (e.g., fuzzy clustering), psychology (e.g., sentiment analyses), or clinical trials (e.g., consensus measurement).
• By offering insights on the variety of possibilities to exploit Delphi-based data, we aim to support researchers across all disciplines in conducting Delphi studies and potentially expand and improve the method's field of application.

## Graphical Abstract

**Figure:**
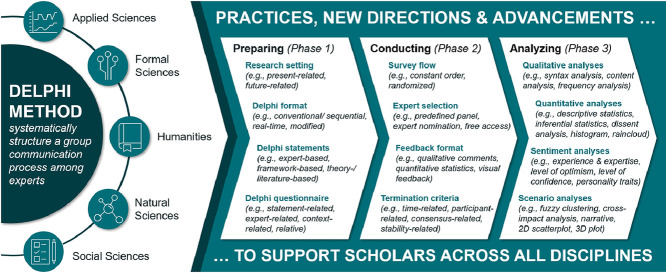

Specifications table

**Table:** 

Subject Area:	Economics and Finance
More specific subject area:	*Decision Sciences*
Method name:	*Delphi Method*
Name and reference of original method:	*Dalkey, N., & Helmer, O. (1963). An experimental application of the Delphi method to the use of experts. Management Science, 9(3), 458–467.*
Resource availability:	*- Delphi survey software (e.g., Mesydel, Millennium Project RTD, Surveylet, Welphi)* *- Classic survey tools, in case sequential Delphi rounds use separate surveys (e.g., SurveyMonkey, Qualtrics)* *- Software supporting qualitative data analysis (e.g. NVivo, Atlas.ti)* *- Microsoft Office (e.g., Word, Excel, or Latex)* *- Statistical software (e.g., R, STATA, or SPSS)*

## Method Basics and Co-Submitted Research

### Basics of the Delphi study

The Delphi technique is a scientific method to organize and manage structured group communication processes with the aim of generating insights on either current or prospective challenges; especially in situations with limited availability of information [21,^48^,^74^,77]. As such, it has been frequently used in various scientific disciplines ranging from health care [14,^29^,^51^,62], medicine [24,^43^,^63^,86], education [15,^72^,88], business [19,^95^,98], engineering and technology [11,82], social sciences [10,89], to information management [4,81], and environmental studies [83]. Irrespective of the focus in time or content, the Delphi technique builds on the anonymity of participating experts who are invited to assess and comment on different statements or questions related to a specific research topic [47,59]. Quantitative assessments traditionally include probability, impact, and desirability of occurrence, but are not limited to these. Further dimensions could refer to innovativeness, urgency, or (technical) feasibility, for instance. Moreover, participant-related information such as confidence or expertise can be collected [25,^32^,87]. In addition, especially in medical and clinical research, Delphi studies make use of rank-order questions, rating scales, or open questions, while often being designed to examine levels of consensus among experts [14,^72^,86]. In a Delphi survey, the aggregated group opinion is fed back to participants across multiple discussion rounds of the same set of theses. During this multi-round procedure, the rounds can be performed sequentially, or – with the help of dedicated software – immediately (so-called real-time Delphi) [2,^35^,36]. After each round, panelists have the possibility to review the aggregated results and to reconsider their assessment based on the added quantitative and qualitative information [12,53]. This structured group communication process is supposed to lead to a convergence – or divergence – of opinions, hence, producing more accurate results than traditional opinion-polling techniques [59]. Moreover, the Delphi method has advantages over in-person techniques such as group discussions or brainstorming sessions, as it rules out personal sensitivities among the experts and therefore avoids potentially destructive group dynamics [99]. The results of a Delphi survey can deliver stand-alone insights but are increasingly linked to scenario analytics, to fulfill idea-generation, consolidation, or judgment functions [68].

### Co-submitted research

The technical paper at hand builds on a Delphi study including scenario analysis, which is dealing with the impact of COVID-19 on the European football ecosystem [8]. The study included 110 international experts and was conducted amid the COVID-19 outbreak between April and May 2020. In times of deep uncertainty, participants evaluated the regulatory, economic, social, and technological implications of the pandemic on the European football ecosystem [97]. In this context, the study served two main purposes: on the one hand, it facilitated an expert discussion that was valuable for all participants as they faced a similar level of unprecedented ambiguity and thus shared common challenges. On the other hand, it aimed to advance the Delphi technique from a methodological point of view by offering a comprehensive analysis and combine cross-disciplinary features. For example, the authors conducted dissent analyses from the field of risk and emergency preparedness, while introducing a sentiment analysis of the field of psychology. The latter was of particular importance in times of crisis in order to interpret the experts' assessments against the backdrop of their individual situation or constitution. All in all, the Delphi method proved to be a suitable technique to manage a systematic online dialogue among experts while at the same time assuring scientific rigor to derive accurate results.

### Structure and aim of this paper

**Fig. 1:**
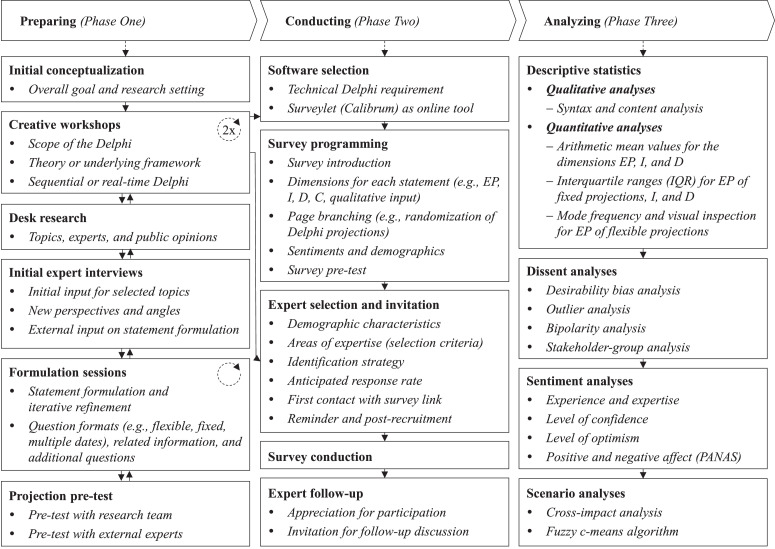
Three Phases of Delphi-based Research

We structure this technical paper following the three major phases of a Delphi-based research project: preparing, conducting, and analyzing. Each phase consists of different steps (as depicted in Fig. 1), which will be thoroughly explained in this paper. In this context, we provide a comprehensive overview of potential features and recent advancements in all three phases (see Table 1, [5], [6], [17], [23], [26], [28], [30], [31], [37], [39], [41], [46], [55], [57], [67], [64], [65], [79], [85]) and therefore complement and substantially extend recent methodic publications such as Schmalz et al. [80]. Thereby, we aim to support the research community in utilizing the Delphi technique for their respective disciplines by following a replicable, but still highly customizable approach.

**Table 1:** Overview of Potential Delphi Features.

## Preparing a Delphi study (Phase One)

**Fig. 2:**
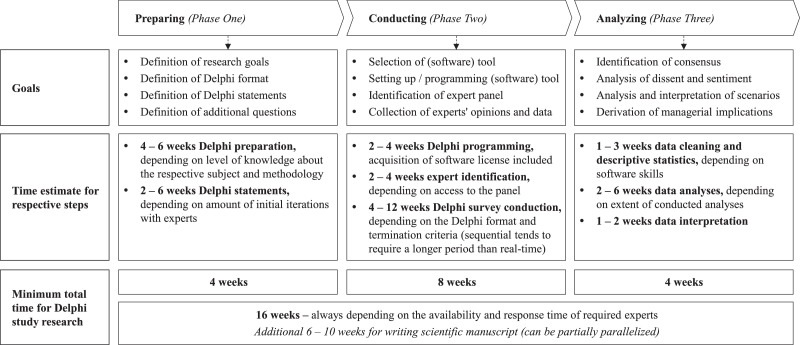
Goals and Time Estimates for Delphi Phases

Thorough preparation is critical to ensure the validity and accuracy of a Delphi study [45,80]. In general, this phase pursues four different goals: (1) *Definition of research goals*, (2) *definition of Delphi format*, (3) *definition of Delphi statements*, and (4) *definition of additional questions* (see Fig. 2). To achieve these goals, we started with an initial conceptualization phase followed by two creative workshops in order to define our *research goals* and the *Delphi format*. Simultaneously, we conducted desk research to understand the current body of research and to identify the major challenges in the industry. Given the topicality of events around the pandemic, the existing body of research on the impact of COVID-19 on sports industries was scarce. Therefore, we decided to involve experts early in the process to define our overarching topics and thus our *Delphi statements*. To refine these, we conducted 17 formulation sessions with the research team and fed back the proposed statements as well as *additional questions* to our experts. Eventually, we also tested our statements with previously not involved researchers and experts to ensure the comprehensibility of our statements. To allow the research community to thoroughly understand and adapt this research process, we will describe each step in more detail below.

### Initial conceptualization

The initial conceptualization was necessary to define the overarching *research goal*, which – in our case – was twofold. On the one hand, we wanted to facilitate an expert discussion in the European football industry amid the COVID-19 crisis to thus provide practical added value to all participants who faced unprecedented challenges due to the pandemic. On the other hand, we wanted to gain accurate insights on the short-, mid-, and long-term effects of COVID-19 on European football by conducting a state-of-the-art Delphi study. To achieve these two goals, we compiled a research team with expertise in terms of content (i.e., European football) as well as methodology (i.e., Delphi technique). Given the urgency, we also developed a tight timeline for preparing and conducting our research – with roughly 5 weeks from initial conceptualization in mid-March 2020 to the actual survey launch in mid-April 2020.

#### Technical recommendation for “2.1 Initial conceptualization”

• We deliberately included researchers with different expertise in terms of industry specifics, methodological experience, and statistical knowhow. We made good experience with this composition and motivate researchers to include methodological and statistical expertise in the research team as this significantly accelerates the research process.
• We encourage scholars to put dedicated effort into defining a research direction as well as reviewing and assessing the suitability of the Delphi technique and potential software solutions.

### Creative workshops

The creative workshops were used to define the *Delphi format*. For us, the *Delphi format* includes three central elements: *(1) scope, (2) theory/framework,* and *(3) sequential or real-time conduction.* In terms of *scope,* we decided to focus on the European football ecosystem both because we wanted to include experts from different backgrounds, organizations (i.e., clubs, leagues and associations, academia, football-related adjacencies), and from all of the five core European football markets (i.e., Germany, United Kingdom, Spain, France, Italy). This helped us to cover a broad range of perspectives and allowed us to get an international perspective on the impact of the pandemic.

From a *theory/framework* perspective, we conducted a literature review to identify an adequate structure on which we could base our research. To cover a wide range of potential effects of COVID-19 on the European football ecosystem, we decided to build on the PEST framework (political, economic, socio-cultural, technological) [42] and extended the political dimension with a regulatory perspective, which appeared to be more suitable in the context of football, so that we introduced the REST framework (Regulatory, Economic, Social, Technological) for our context [61,68]. In our second workshop, we discussed this framework with five previously not involved industry experts to obtain additional and unbiased perspectives. As a result, we decided to split the economic angle of our REST framework into two separate buckets focusing on revenue-related and cost-related economic effects. This modification towards a REEST structure (Regulatory, Economic – revenue, Economic – cost, Socio-cultural, Technological) helped us to refine our *Delphi format* by putting more emphasis on the economic pressure that many football-related organizations felt during the first lockdown in April 2020 and therefore increased the relevance of our study. We encourage researchers to not blindly follow existing frameworks but to adjust them to their needs as appropriate.

The decision for a *sequential or real-time Delphi* was made in favor of the real-time format due to the ambitious timeframe of the actual survey conduction and due to the improved user experience for participants, which often results in higher participation and lower drop-out rates [2,36]. For a more detailed discussion on decision criteria between sequential or real-time Delphi see Gnatzy et al. [35].

#### Technical recommendation for “2.2 Creative workshops”

• Defining a framework for the initial list of Delphi statements helped to structure our thinking and to involve experts for different topic areas. In addition, it contributes to the completeness of the set. We therefore highly recommend using a framework to structure and cluster Delphi statements.
• If a framework is not applicable for specific research contexts, it is advisable to anchor the Delphi research based on existing theories. A good example of this is Winkler et al. [100], who used the "organizational information processing theory (OIPT)" as a foundation for their Delphi study to analyze decision making in emerging markets.
• The real-time Delphi format allowed us to involve a larger number of experts in a shorter amount of time. We observed that much more than 50% of the experts joined the Delphi discussion after three weeks of the initial launch and then engaged vividly in the discussion. The sequential approach tends to complicate participation for late joiners so that we would recommend a real-time format in case the schedule is tight.

### Desk research

Dedicated desk research was performed in between and after the two creative workshops. As Schmalz et al. [80] conclude, a thorough literature review is indispensable for a Delphi study. However, this does not necessarily need to be limited to the scientific body of research – particularly in the case of prospective, forecast studies for which existing literature might be scarce. In the special case of our co-submitted research, for example, there was almost no existing research on the consequences of COVID-19 at the beginning of the crisis. Therefore, we also focused on the popular press to identify the most urgent issues for the European football ecosystem. To do so, we screened international newspapers and pertinent sports management magazines to get a first idea for potential *Delphi statements*. This initial long list of statements was captured in Microsoft Excel and shared with the five above-mentioned experts who participated in our second workshop. Their input was used to further expand the statement long list which then served as a basis for our initial expert interviews.

#### Technical recommendation for “2.3 Desk research”

• The desk research should be structured based on a framework or theory, too. The lack of a proper foundation jeopardizes not only the quality of desk research but also the comprehensiveness of the literature review that should be part of all scientific publications.

### Initial expert interviews

Based on the modified REEST framework, we decided to conduct three initial expert interviews for each of our five framework dimensions, following a semi-structured approach [1]. The panel was meant to represent all stakeholders within the European football ecosystem, which is why we interviewed subject matter experts from all five European target countries as well as the four stakeholder groups. In total, we contacted 21 experts via email or directly via phone and achieved a final response rate of 71 percent. We recommend activating contacts from (wider) personal networks to increase the response rate and to speed up the process so that two weeks from first inquiry to final interview becomes a realistic target.

We scheduled all interviews for 60 min and spent roughly 15 min explaining our *research goals* as well as the characteristics of a Delphi survey. We then spent 30 min discussing the main challenges caused by COVID-19 for the expert's respective area of expertise and developed/refined potential *Delphi statements* and saved the last 15 min for open questions and follow-up information. The latter included an invitation to the actual Delphi survey as well as an inquiry to nominate a list of potential experts as proposed by Belton et al. [9]. After each interview, members of the research team reviewed the findings and conducted formulation sessions, which are described in the next section. The results of these sessions were then used for the next interview so that we iteratively developed our *Delphi statements*. At the end of the process, we shared the short list of statements in Microsoft Excel with all experts and received their proposed prioritization which helped us identify our final set of 15 statements.

#### Technical recommendation for “2.4 Initial expert interviews”

• We made a very good experience involving experts outside the research team early in the process. However, the amount of 15 initial experts was surely the upper limit. For most prospective Delphi studies, 5 to 8 initial experts should be sufficient.
• Contacting experts from the closer network, accelerated our process significantly, since we were able to speak to most experts on short notice with a very high response rate. However, research teams should bear in mind potential biases and homogeneous thinking within the close network.

### Formulation sessions

The accurate wording of statements is central to the quality of Delphi studies as it can reduce biases and increase response variance [27,57]. Therefore, we conducted regular formulation and review sessions (17 iterations in total) with at least two participants (one permanent and four alternating research team members). This setup guaranteed that the core research team member was aware of all information while being challenged by others in terms of subjective biases [101]. The goal of our formulation sessions was not only to define the final set of *Delphi statements*, but also to decide on question formats, related information, and *additional questions*.

For the formulation of *Delphi statements*, we followed the guidelines by Markmann et al. [57] and iteratively shaped the wording with our experts. To balance the trade-off between the gain of insight and participation effort, we included 15 statements (three for each dimension of our REEST framework) in our study. Moreover, we discussed the question format and decided to query the expected probability (EP) of occurrence as our main variable, given the prospective nature of our Delphi. Moreover, we used desirability (D) and impact (I) of occurrence as complementary variables, and confidence (C) in assessing the respective statement as a bias control variable. For the dimensions D, I, and C we chose a traditional five-point Likert scale from very low (1) to very high (5). The EP dimension, in turn, can have different question formats, such as fixed formats (e.g., Liker-scale, or 0–100 percent scale to assess the expected probability of occurrence by a certain time) or flexible formats (e.g., assessment of time when occurrence is most likely, or assessment of expected probability of occurrence at several points in time in the future). For the co-submitted research, we decided to mix fixed and flexible statements, because we wanted to have both a focus on short-term effects of COVID-19 (which we tested with fixed statements with the end date 2022, e.g., *``in 2022, (strategic) investors got more shares in European football clubs due to COVID-19´´*) and an indication for medium- to long-term consequences of the pandemic (which we tested with flexible statements, e.g., *``A salary cap for professional football players has been introduced´´*). We also discussed relevant information associated with our statements and decided to present two exemplary pro arguments as well as two exemplary contra arguments for each statement as initial conditions [35]. This information provided a common basis for all experts and was supposed to motivate participants to think of both supporting and opposing arguments, which is a way to mitigate biases such as framing, anchoring, or desirability bias [13]. For the same reason, we asked participants to separately share qualitative comments in favor and against the occurrence of the respective statement. To gain further insights, we also included an open-comment option for the impact of occurrence. To keep the survey length reasonable, we decided to dispense free-text fields for the desirability and confidence dimensions.

Last, we used the formulation sessions to agree on *additional questions*. These included classic demographic questions such as gender, age, country of residence, type of organization, and years of work experience within the European football industry. In addition to these surface-level criteria, we also asked for deep-level characteristics, because we wanted to learn about the values and beliefs of participating experts, which might affect their opinions [56,87]. These consisted of the respective area(s) of expertise (e.g., strategy, sponsoring, marketing, digital, legal) as well as personality-related information [56]. The latter included COVID-19-related questions to assess experts' level of optimism and a short version of the positive affect negative affect scale (PANAS) to judge on experts' sentiments [78,^91^,97]. In conclusion, the formulation sessions eventually determined the *Delphi format, Delphi statements*, and *additional questions*, which is why we want to emphasize the importance of this step within a Delphi research project.

#### Technical recommendation for “2.5 Formulation sessions”

• While we conducted 17 formulation iterations, we think that fewer sessions would be sufficient. Also, these formulation sessions can be very informal.
• We recommend involving at least 3 researchers in the process of formulation in order to avoid subjective perspectives and biases.
• The mix of fixed and flexible statements complicated subsequent analyses significantly (as described in the respective lessons learned in the analysis section). Therefore, we recommend sticking to consistent scales for each individual dimension of assessment (i.e., expected probability, desirability, and impact).
• Three open-text questions per Delphi statement seemed to be the maximum for our amount of 15 statements. We experienced that responses in the impact-related commentary field often referred to aspects that were mentioned in the probability-related comments before. Therefore, we argue that one or two open-text questions per Delphi statement are suitable to get enough qualitative input, while not risking increased survey fatigue.
• We understand that in other disciplines qualitative feedback might not be at the core of investigation. Therefore, also larger amounts of statements (up to 100) with few questions (e.g., importance and relevance) can be suitable [15].
• We made a good experience illustrating only one Delphi statement per webpage, in order to avoid the necessity to scroll online. From our experience, this style of presentation prevented experts from overlooking free-text fields and allowed participants to get accustomed to a consistent format. In order to guide participants through the survey, we added an overall progress bar and included a "half-time message", indicating that 50 percent of the survey was completed.
• We highly encourage researchers to include additional questions, because they can help to learn more about experts' personal predispositions. For more details see section ``4.3 Sentiment Analysis´´.

### Survey pre-tests

In between the last two formulation sessions, we selectively pre-tested our *Delphi format, Delphi statements,* and *additional questions* with fellow researchers and experts from the creative workshop in order to ensure clear comprehensibility and guarantee high reliability [69,70]. Based on these pre-tests, we slightly adjusted our final wording. In the co-submitted paper itself, we referred to our *Delphi statements* as *Delphi projections*, which is particularly common in the context of foresight. For the remainder of this technical paper, we stick to the broader expression of *Delphi statements*.

#### Technical recommendation for “2.6 Survey pre-tests”

• While we put the main focus of our pre-tests on the content of our study, we would highly recommend researchers to also pre-test the average time to complete the survey, as survey length is known to be a critical factor with regard to survey fatigue and elevated drop-out rates [38]. Based on feedback from participants we learned that our survey length was about 45 min and therefore at the upper limit for the context of our field of study. However, reasonable durations might be shorter or longer for other settings, which should be pre-tested with representatives of the respective expert panel.

## Conducting a Delphi study (Phase Two)

In terms of the actual conduction of the Delphi survey, this technical paper will focus on software selection and programming as well as the identification and interaction with experts. To the best of our knowledge, real-time Delphi software has only been applied in business and forecasting studies so far. We encourage scholars of all other disciplines to consider such applications during the survey design in future research endeavors.

### Software selection

As mentioned earlier, we decided to conduct a real-time Delphi in order to account for the ambitious timeframe and to allow participating experts to review the most recent results at any point in time. In general, we advise defining the type of Delphi (i.e., sequential or real-time) early in the research process. Based on the respective *research goals*, one or the other type might be more suitable. While web-based software is strictly required for real-time Delphi surveys, sequential studies can still be distributed via mail or even phone; although this is rather an exception nowadays [14]. In terms of web-based software, Aengenheyster et al. [2] compared state-of-the-art providers regarding features, data output, user-friendliness, and ease of administration. Based on their assessment and our own market screening, we decided to choose *Surveylet* as our preferred platform. The provider, *Calibrum*, offers different service packages, which range from pure platform access to full-service support. For the co-submitted research, we acquired a medium package including basic service support and individualization options.

#### Technical recommendation for “3.1 Software selection”

• Surveylet offers a variety of options, which might even go beyond the relevant set of functions for most Delphi studies. It also allows for individualization options and service support, which typically require more expensive contracts. Thus, we recommend checking occurring costs. Setting up an account takes roughly one week and should therefore be initiated sufficiently earlier than the actual survey programming.
• Meanwhile, there might also be additional online Delphi platforms available beyond the scope of the review of Aengenheyster [2]. A more recent example is the BOHEMIA Delphi (Beyond the Horizon – Foresight in Support of the Preparation of the EU‘s Future Policy in Research and Innovation) [34].

### Survey programming

While software selection and preparations can be performed early in the process, we highly recommend finishing phase one (i.e., definition of *Delphi format, Delphi statements*, and *additional questions*) before starting the actual survey programming. Subsequent changes to format and statements lead to extra effort and significantly increase the error-proneness. Therefore, we captured and refined all relevant text modules in Microsoft Excel, prior to programming the survey. These included the survey introduction, the actual statements, pro and contra arguments, as well as all additional questions and an outro.

Special attention should be paid to the survey introduction, particularly in web-based Delphi studies, as a proper understanding of the process is crucial for panelists. We recommend a short, but very concise introduction, including *(1) the purpose and anticipated duration of the study, (2) contact details of the research team,* and *(3) information about the Delphi process*. For the explanation of the Delphi process, we recommend mentioning the anonymity of participants and the iterative character of the method. In this context, we encouraged participants to also share (and review) qualitative comments. Moreover, we explicitly draw attention to potential biases, that might affect participants' evaluations. By addressing these issues, we aimed to sensitize participants to deliberately avoid these biases [13]. Eventually, we offered a link to a short online tutorial (approximately 90 seconds), that explained the overall Delphi process with visual support.

In terms of *Surveylet* as the software of choice for our co-submitted research, we made good experience with the following settings and programming steps: First, we recommend tracking all possible statistics, which include more than 30 variables such as mean values, standard deviations, and interquartile ranges. These should generally be displayed to the survey administrator and can selectively be displayed to the participants. While more data result in more information for the experts, they can also trigger biases, so that we decided to only share mean values with our participants [13]. We refrained from using real-time text analyses, as these were – at the time of our survey – not fully mature, causing significantly longer loading times of the website. An option that appeared quite useful to us was the randomization of statements. That is, every *Delphi statement* along with the related questions was presented in randomized order, which prevented the risk that experts put more effort into early statements or get collectively biased due to previous answers.

#### Technical recommendation for “3.2 Survey programming”

• When it comes to survey programming, we recommend enough preparation time (at least 2 weeks). For novice users, we also recommend basic service support for the first Delphi study or more preparation time to understand the most important features.

### Expert selection and invitation

The initial identification of experts can be a challenging task, depending on the subject that is supposed to be explored [25,32]. Based on the existing body of literature and the experience from our co-submitted research, we suggest considering five aspects when composing a Delphi expert panel: *(1) Size of the panel, (2) level of expertise, (3) level of heterogeneity, (4) level of interest,* and *(5) access to the panel*.

While the specific context of investigation will surely have an impact on the panel composition, it is always advisable to address all five aspects early in the process. In our co-submitted research, we wanted to gain an understanding of prospective developments and aimed to include different stakeholder groups to obtain a comprehensive view of an entire ecosystem. Therefore, the *size of the panel* needed to be rather large. In general, we recommend a larger number of participants for more holistic topics (as often found in management research) and a more condensed set of experts for specialized topics (as often found in the clinical context). For statistical purposes, it is advisable to have at least 15 to 20 experts in any given sub-group of experts, if significant differences between these sub-groups are supposed to be statistically analyzed. Moreover, we learned that the variety in additional qualitative comments typically decreases from a quantity of 30 to 40 participants. Similar to the size of the panel, the *level of expertise* depends on the subject. While there might be a need for specific domain knowledge in some cases, other Delphi surveys might benefit from a broader more generalist perspective of participants. In any case, it is necessary to predefine criteria for level of expertise, such as age, years of work experience, occupation, academic degree, or the number of publications in a certain field of research. These criteria then help to justify the panel selection and potentially allow to distinguish between groups based on expertise. Another important aspect of panel composition is the *level of heterogeneity*. Especially in more holistic – often future-related – settings, a heterogeneous sample can mitigate cognitive biases [13]. Moreover, a variety of backgrounds offers room for inter-group analyses. Possible categories for preselection include dedicated experts from academia, politics, the broader public, and obviously the specific industry that is supposed to be evaluated. Based on our past experience, we also encourage researchers to assess the *level of interest* that certain participants might have with regard to the survey results while bearing the risk of a potential self-selection bias in mind [40]. Time and attention of subject matter experts are scarce and therefore personal investment of participants can increase response rates and quality of comments. Similarly, *access to the panel* should be evaluated early in the process. While there are always experts for each and every topic, it is not always easy to reach out to them directly.

To invite experts, the software tool *Surveylet* offers a variety of options. Based on the *size of the panel,* we recommend either pre-populated links (i.e., one individual link for each participant based on the participant's e-mail address) for smaller panels with available contact details or in case of larger panels an open link, in which each expert has to insert his or her e-mail address as a unique identifier. At this point, it is important to assure participants that the e-mail address purely serves as an identifier to revise previous inputs.

#### Technical recommendation for “3.3 Expert selection and invitation”

• A first and crucial step is to define the criteria on how to measure expertise for the research endeavor. This holds especially true for Delphi studies, where it is rather not about the representativeness of a population but the identification and inclusion of the highest-level of expertise in the panel. A systematic review of expert identification methods can, for example, be found in Mauksch et al. [58].
• Our goal was to include more than 80 participants in our survey because we wanted to differentiate between four sub-groups of experts (with at least 20 participants per sub-group). We argue that for a holistic prospective, forecasting Delphi survey with at least three sub-groups of experts, a quantity of 80 participants is sufficient. With a conservative average response rate of 10% (we had 16.2% in our co-submitted research), this would require an initial set of 800 invited experts, which might already be a prohibitively high number for some fields of research. In these cases, we recommend aiming for smaller samples and more focused statements.
• For larger samples, we recommend examining the panel composition on a regular basis during the survey. If necessary, it can be helpful to additionally invite targeted experts to ensure balanced sub-groups of experts (e.g., from industry, politics, and academia).
• For smaller samples, we recommend creating individualized links (if applicable), because this offers maximum convenience to participants. We used such links to invite our initial experts to participate in the survey and also created individual links if we had the respective contact information.
• Our chosen Delphi software allowed adjusting the "landing page" by altering the URL. In this specific case, we highly recommend asking for participants’ e-mail addresses only. On the one hand, this allows users to not share their full names. On the other hand, the e-mail address serves as a unique identifier for the platform, thus allowing participants to access the survey from different devices. If the e-mail address is not requested, participants can only review their given responses, if they use the same device (and did not clear their cache).

### Survey conduction

With regard to the actual survey conduction, we recommend an a priori definition of (cascaded) termination criteria. Typically, termination criteria are either time-related, participant-related, or consensus-related. Time-related criteria might include the number of rounds for sequential Delphi studies, or a certain time period for real-time Delphi studies [26]. Participant-related criteria could refer to the number of experts that participated in the study and – within the real-time format – revisited the survey at least once. If the Delphi study addresses consensus, also dedicated measures such as agreement thresholds (e.g., interquartile range, mode frequency), or stability measures (e.g., coefficient of variation, nonparametric χ² test) can serve as termination criteria [7,94]. Particularly with regard to the set of stability and agreement criteria, Dajani et al. [20] proposed a theoretical hierarchical model to stop or adjust the Delphi process. Von Briel [92] and Culot et al. [18] represent examples of this approach.

While there is a common notion that Delphi studies in principle follow a consensus-building purpose, we argue that similarly, disagreement among experts is a valid and very insightful outcome, especially in prospective studies. Therefore, we applied a cascaded termination logic with agreement and stability thresholds on the first level and a time-related criterion (maximum 8 weeks) on the second level. Since we did not reach consensus on all statements after 8 weeks, we terminated the survey and included all participants who re-visited at least once in our analysis. Over the course of our survey period, we sent out reminder emails twice: After 3 weeks we contacted all experts that had not yet participated and after 6 weeks we sent a reminder to all participants who answered the survey and asked to review and revise their inputs. For this purpose, our selected Delphi software offered a function to address different groups of participants (e.g., based on their progress within the survey) separately, which can be a helpful service.

#### Technical recommendation for “3.4 Survey conduction”

• Given our real-time format with more than 100 participants, it was difficult for participants to grasp all qualitative inputs shared by their peers. To help participants distinguish between pro and contra arguments concerning the expected probability of statements, we included two separate text boxes. However, there is always a trade-off between the amount of requested information and required time spent by the experts. Thus, we recommend using less qualitative input fields for more practical-oriented studies.

### Expert follow-up

In order to inform all participants about our initial results, we shared an overview of our descriptive statistics 6 weeks after the termination of the survey. In doing so, we aimed to enrich the practical discussion without having to wait for the scientific publication, which typically consumes several months including revisions. While this step is particularly important for urgent topics, we generally recommend some kind of expert follow-up in order to appreciate the time and effort that participants put into the study.

#### Technical recommendation for “3.5 Expert follow-up”

• There is a risk of revealing results before analyzing them thoroughly. Therefore, we decided to share descriptive statistics after we completed the analyzing phase, but well before the actual research paper was published. To reward participants for their time and effort, we would highly recommend sharing basic results as early as possible. This is particularly important in the context of urgent and up-to-date topics.

## Analyzing a Delphi study (Phase Three)

The possibilities of analyzing Delphi-based datasets are manifold. In our co-submitted research, we split our analyses into four different categories: *(1) Descriptive statistics, (2) Dissent analyses, (3) Sentiment analysis,* and *(4) Scenario analysis*. To analyze our dataset, we used the open-source software *R*. We made a very good experience with this software because it allows conducting almost any relevant analysis with publicly available software packages.

### Descriptive statistics

Descriptive statistics of Delphi-based datasets typically include qualitative and quantitative analyses. We also motivate researchers to include a post-hoc Mann-Whitney *U* test at the beginning of the descriptive statistics to check for non-response bias [84].

### Qualitative analyses

Qualitative analyses particularly focus on experts' comments and can reveal insights about the participants' level of engagement as well as potential interrelations between different *Delphi statements*. For data type transparency, we highly recommend conducting a *syntax and content analysis* as suggested by Förster and von der Gracht [32]. In terms of syntax, we labeled all comments as either whole sentences, phrases, or catchwords. A high percentage of whole sentences generally indicates a solid level of engagement in the discussion and should therefore serve as a quality measure [73]. To analyze content, we had two researchers coding the comments as beliefs, differentiations, cause-effect relationships, examples, historical analogies, experiences, trends, figures, no information, or misunderstandings. To assure concordance, we calculated the level of agreement between the two coders. With an agreement rate of more than 80%, we inferred acceptable interrater reliability [54].

To gain further insights from participants' comments, we recommend performing a *cross-impact analysis* (for additional illustration, see e.g., [6,71]) in order to understand potential interaction effects between statements. Therefore, we assessed the active and reactive effects among our statements by considering the results of our content analysis. We then plotted the results and categorized statements as buffering (limited active or reactive effect), active, reactive, and critical (strong active and reactive effect) statements. These insights helped us to interpret our results in the scenario analysis and validated our effort to formulate largely independent *Delphi statements*.

### Quantitative analyses

For our basic quantitative analyses, we calculated *arithmetic mean values* and *standard deviations* for our three statement-related dimensions expected probability, impact, and desirability. To assess consensus, we used *interquartile ranges* due to their robustness as a statistical measure. While there are multiple interpretations in literature, we argue that a threshold of a maximum of 25% of the respective scale (e.g., 25 on a scale from 0–100, or 1.25 on a scale from 1–5) can serve as an indicator for consensus. For our flexible projections, in turn, we utilized *mode frequency* and a visual inspection of *histograms* to infer information about consensus, or potential dissent schemes, as explained in the subsequent section.

#### Technical recommendation for “4.1 Descriptive statistics”

• Although time-consuming, we made a good experience with two coders for all qualitative analyses. Insights from participants' comments are a valuable input for the analyses and discussion. Although often underreported in many Delphi-based journal articles, we argue that the qualitative part of the methodology should not be ignored.
• Despite the fact that there are various quantitative measures for consensus and stability, we made good experience with interquartile ranges as a measure of choice. Alternative approaches (e.g., fuzzy statistics) can be suitable under special circumstances, but would not have revealed extra insights in our case [16].

### Dissent analyses

The major aim of the Delphi method is to systematically structure a group communication process [53]). This process might lead to consensus, but as with for example Policy-type Delphi studies (see e.g., [22]), researchers could be more interested in the dissent of the panel. Especially for prospective studies, we argue that dissent can reveal valuable insights for the practical and academic discussion. Therefore, we present how we applied a series of potential dissent analyses in our co-submitted research, which were initially introduced by Warth et al. [96].

#### Desirability bias analysis

In many forecast surveys, participants tend to assess desirable developments as more likely than undesirable ones [101]. Therefore, we tested for a potential desirability bias, following the approach presented by Ecken et al. [27]. It includes a post hoc adjustment of expected probability values based on the desirability assessments of experts. As the calculations for this method require a restructured dataset in the long format (i.e., one row per participant per statement), it takes quite a lot of effort. Based on our experience, we would recommend using a less time-consuming technique to account for a potential desirability bias (e.g., by partializing out the influence of desirability on expected probability, or by conducting simple correlation analyses).

#### Outlier analysis

Outliers can have a significant effect on statistic variables, such as the interquartile range [3]. Therefore, we identified and eliminated outliers to test if these had an impact on the group's consensus. In our co-submitted research, we found no significant effect, however, we would recommend running this analysis and interpreting the results. Especially if the respective outliers shared out-of-the-norm qualitative comments, these might deliver valuable insights. Alternatively, they could also point towards (systematic) misunderstandings, which would be a potential reason to either delete the specific participant from the dataset, or to double-check the comprehensibility of the specific statement.

#### Bipolarity analysis

The bipolarity analysis accounts for the fact that there might be opposing groups of experts with respective intra-group consensus [77]. To test for this effect, we checked for bimodal distributions and visually inspected histograms of expected probability assessments for all statements. While we had little indication for strong bipolarity in our co-submitted research, this simple analysis should always be conducted as part of the result evaluation. Bipolarity – if present – almost prohibits consensus. Therefore, it is even more important to study the two extremes to understand if these are close together or rather far apart from each other. Either constellation could reveal valuable insights.

#### Stakeholder-group analysis

A classical dissent analysis that can be found in multiple disciplines is the stakeholder-group analysis. For the co-submitted research, we distinguished four stakeholder groups based on their occupation. To identify opposing views, we conducted Mann-Whitney *U* tests between the four groups for all 15 statements and reported (marginally) significant differences between groups. Although this analysis requires substantial time effort, it is fairly easy from a methodological point of view and we highly recommend differentiating stakeholder groups, as it provides valuable insights with practical relevance.

#### Technical recommendation for “4.2 Dissent analyses”

• We see consensus and dissent analyses as two sides of the same medal. Although both directions might ask for different analysis steps, measures, and thresholds, we recommend applying both perspectives to the Delphi dataset. Especially for dissent, which is often neglected, we see high value and additional insights in analyzing the potential reasons for diverging opinions.
• When planning the expert panel, it is advisable to think about potential stakeholder-group analyses early in the process. To obtain reliable results and bearing statistical requirements in mind, each subset should consist of at least 15 to 20 participants. This should be considered in the panel composition.
• In addition to analyzing pre-defined stakeholder groups, an explorative group analysis based on participants’ assessment patterns inherent in the data could be beneficial. There might be strong dissent across identified groups such as technology optimists, sustainability pessimists, or transformation skeptics.

### Sentiment analyses

While the importance of considering participants' sentiments in prospective studies was pointed out in the 1980s, especially the personality dimension is rarely found in Delphi-based studies in the past decades [56,87]. However, detailed information about the personality of participants can shed a different light on results and should therefore be considered in all Delphi studies, irrespective of the individual discipline. While there is a myriad of possibilities to cover personality and expert-related information, we covered four dimensions for sentiment analysis: *(1) Expertise and experience, (2) Level of confidence, (3) Level of optimism,* as well as *(4) Positive and negative affect*.

#### Expertise and experience

To assess expertise and experience, we asked for our experts' years of professional experience within the industry we examined. Based on this information, we calculated correlations between years of experience and expected probability assessments and reported significant effects. Moreover, participants were able to indicate their knowledge in specific topic areas such as strategy, marketing, sales, and digital. This served as the foundation for subset comparisons, similar to the stakeholder-group analysis.

#### Level of confidence

**Fig. 3:**
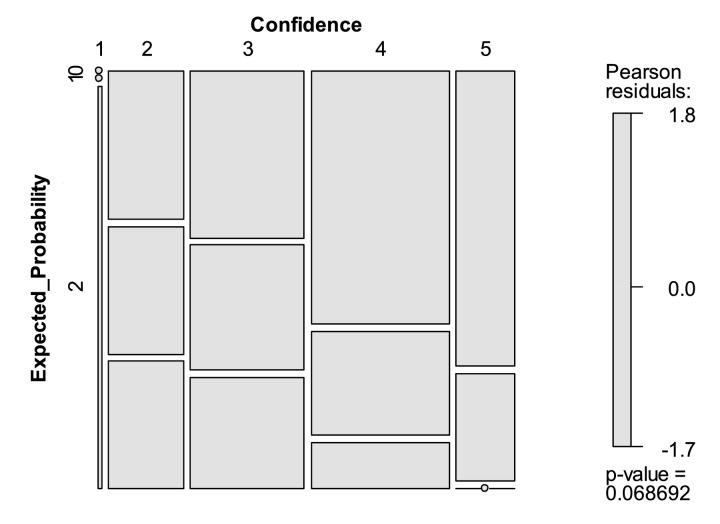
Visualization of Relationship between Confidence and Expected Probability. Note. This mosaic plot is based on the data of Beiderbeck et al. [8]. It shows the relationship between confidence (measured with a five-point Likert scale from 1 = very low to 5 = very high) and expected probability (0 = statement will never occur; 1 = statement will occur long-term; 2 = statement will occur short-term). Size of the respective mosaic represents number of participants with respective confidence and expected probability assessment.

In our co-submitted research, we collected information on our experts' subjective knowledge on each statement, by asking for confidence in assessing the respective topic. We used a five-point Likert scale from 1 (very low) to 5 (very high) and then calculated correlations between confidence and expected probability for statements with linear intervals and chi-square tests for statements with non-linear intervals. While we only reported our results verbally, there are also insightful ways to illustrate these analyses, as exemplarily depicted in Fig. 3.

#### Level of optimism

While experience, expertise, and confidence find more frequent application in Delphi-based manuscripts, we rarely find other indicators for deep-level expert characteristics in use [87]. Therefore, we included the level of optimism as an indicator for a personality trait that is relevant for future predictions [56]. We posed two dedicated questions with respect to the overall future developments within the industry of investigation. Based on the responses we conducted a median split and created two subsets of rather optimistic and rather pessimistic experts. We then conducted Mann-Whitney *U* tests and reported significant differences between these two groups.

#### Positive and negative affect

Given the circumstances of the COVID-19 outbreak, we also wanted to account for the subjective wellbeing of our experts, which might have affected their respective assessments. Therefore, we used a shortened version of the PANAS (Positive and Negative Affect Schedule) [91] and asked experts to evaluate a four-item construct for two points in time: prior to the crisis and during the crisis. This helped us to calculate differences to see which expert was more or less affected by the pandemic in terms of his or her subjective wellbeing. Again, we used this information to calculate correlations and present significant effects.

#### Technical recommendation for “4.3 Sentiment analyses”

• Instead of "level of optimism" it could also make sense to test for other deep-level characteristics of participants. These can be adapted based on the respective field of research. In the context of technology forecasting, for example, it could be worthwhile to test related constructs such as the trust in technology [60], affinity for technology interaction [33], or attitudes towards using technology [90]. In this context, the inclusion of underlying theories like the technology acceptance model [52] might also be an interesting avenue for future research.
• To account for the individual wellbeing of participants there are numerous alternatives to PANAS, such as the "profile of mood states" or "circumplex model of affect" [75]. While these constructs allow for a more nuanced differentiation of affect, we still decided to use PANAS, because it offered a four-item short version and therefore mastered the trade-off between quality of insights and amount of effort for the participants.
• Another dimension of the sentiment analysis could be "locus of control" – which also offers a short version – to determine participants' perceptions on heteronomy and self-determination [50].

### Scenario analysis

Particularly for prospective studies, Delphi-based insights can serve as a basis for scenario analyses [68]. While there are multiple ways of building and illustrating scenarios, we decided to apply the *fuzzy c-means algorithm* for our co-submitted research. With a significantly high number of experts and assessments, this method yields feasible results. Moreover, it is relatively easy to execute and visualize with *R*. In the case of smaller samples, *hierarchical clustering* might be more appropriate [49]. With dedicated software, the *latent class analysis* offers a further possibility to generate related groups of statements [96].

With the help of the c-means algorithm, we created three groups and plotted the clusters on a 3D coordinate system with the axes expected probability, desirability, and impact to gain a visual impression of our results. In general, we recommend 3D visualizations, because they help the reader grasp the interrelation between three (or more) outcome variables.

#### Technical recommendation for “4.4 Scenario analysis”

• To cluster statements, we needed a comparable output for each individual dimension. In this regard, the different output formats for expected probability required us to introduce a new logic in order to transform and unify the two scales. In our case, this aggregation led to a loss of informational value, because the unified scale consisted of three categories only. Hence, we recommend to not change the format of a scale within one particular dimension of the Delphi survey (e.g. expected probability) or to consider a possible transformation logic upfront.

## Conclusion and future research avenues

In this technical paper, we illustrate a comprehensive Delphi preparation, conduction, and analysis process. We offer room for flexibility in adapting the research process for individual needs while sticking to a consistent framework that allows for replicability.

We encourage researchers from all disciplines to use the Delphi technique in order to organize structured expert discussions around both current and prospective challenges in the respective field of study. From a methodological point of view, we want to support the research community by offering technical recommendations from our Delphi study on the impact of COVID-19 on the European football ecosystem. At the same time, we advocate for further innovative developments of the technique, specifically with regard to the role of experts' personality traits, thinking patterns, and situational concomitant. As with any research, scholars should conclude their research articles with a critical limitations section. During our study of literature, we came across the report of Sackman [76], obviously one of the early critical reflections of the Delphi method. Each Delphi study should include a careful elaboration on its validity and reliability (see e.g., [93], section 3.7 for a review on quality criteria in Delphi surveys), while following evolving quality frameworks, such as described in Jünger et al. [44], Belton et al. [9], and Murphy et al. [66].

## Declaration of Competing Interest

The authors declare that they have no known competing financial interests or personal relationships that could have appeared to influence the work reported in this paper.
